# Crossing the canyon of change: Lessons for leading healthcare innovation

**DOI:** 10.1016/j.fhj.2026.100553

**Published:** 2026-08-07

**Authors:** Sabah Janjua, Jonathan Benger

**Affiliations:** a Faculty of Medical Leadership and Management, 167–169 Great Portland Street, 5th Floor, London W1W 5PF, United Kingdom; b National Institute for Health and Care Excellence, 2 Redman Place, London E20 1JQ, United Kingdom; c Moorfields Eye Hospital NHS Foundation Trust, 162 City Road, London EC1V 2PD, United Kingdom

**Keywords:** Leadership, Education, Medical, Diffusion of innovation, Delivery of health care, Organisational culture

## Abstract

Healthcare is entering an era of rapid technological advancement, yet history reminds us that technologies rarely transform care through technical excellence alone. Their success depends on leaders who can inspire people to adopt, implement and sustain change. This article uses the evolution of modern cataract surgery as a compelling case study to bring Kotter's eight-step model for leading organisational change to life. Through the metaphor of crossing a canyon, it shows how Sir Harold Ridley and Charles Kelman turned innovation into the global standard of care by creating urgency, building coalitions, communicating a compelling vision, removing barriers and embedding new ways of working. Their story offers timeless lessons for today's leaders implementing new technologies such as artificial intelligence, robotics and precision medicine. Innovation alone does not transform healthcare. People do. The leaders who help others cross the canyon will determine whether today's breakthroughs become tomorrow's standard of care.

## Highlights

• New technologies improve care only when people adopt, implement and sustain change.
• Leaders enable this by creating the conditions for people to embrace new ways of working.
• Kotter’s eight-step model is one of the most widely used frameworks for leading organisational change.
• This article brings Kotter’s model to life through the metaphor of crossing a canyon, using the evolution of modern cataract surgery as a compelling example of successful change management.
• As healthcare faces a new wave of technological breakthroughs, this article demonstrates how leadership can help today’s advances become tomorrow’s standard of care.

## Introduction

In healthcare, change is not an occasional activity. It is a constant and evolving process. The challenge of leadership is to inspire others and help them to engage, embark and continue the change journey.

That challenge has never been more relevant. Healthcare is entering an era of rapid technological advancement, with artificial intelligence, robotics and precision medicine reshaping the delivery of care. Yet history reminds us that innovation alone rarely transforms healthcare. Technologies become transformative only when clinicians, organisations and systems can adopt, implement and sustain change.

A useful way of visualising this challenge is through the metaphor of crossing a canyon. Imagine standing at the edge of a canyon. On this side lies familiarity. It feels safe because it is known, yet tigers still roam here: the risks to patients that persist when we accept the status quo. On the other side is food and safety: better processes and better care. Everyone wants to live safe from tigers with food to eat, but the canyon is vast and crossing it feels dangerous. Leaders inspire people to build a bridge and support them to reach the other side.

Kotter's eight-step model^1^, one of the most widely referenced frameworks for leading organisational change, provides a practical way of understanding this journey. It describes how successful change progresses from creating urgency and building guiding coalitions to embedding new ways of working into organisational culture. While often presented as a theoretical framework, its principles are perhaps best understood through lived examples; moments where individuals looked beyond the status quo to shift the trajectory of their entire field.

One of the most remarkable examples in medicine is the evolution of cataract surgery^2^, a procedure whose history stretches back more than two millennia. The surgeon Sushruta described in his treatise circa 600 BC a daring act: inserting a curved needle into the eye to push the clouded lens aside, a technique known as couching. This was one of the earliest recorded attempts at functional surgery. It restored glimmers of light for some, but for most it offered only complications or permanent blindness.

Contrast this with today. Cataract surgery is now the most common elective operation performed in the NHS^3^ and has restored vision to millions of people worldwide. Few stories illustrate the successful translation of healthcare innovation into routine care so clearly: from couching’s perilous gamble to modern cataract surgery’s near universal restoration of sight.

So how did the world cross this canyon? The journey offers a masterclass in leading change.

## Create the right climate for change

### Step 1: create a sense of urgency

Before modern surgery, cataract treatment was risky. Two visionary leaders, Sir Harold Ridley^4^, ^5^ and Charles Kelman^6^, ^7^, saw the tigers on this side of the canyon: large incisions with high risk of sight-threatening infection, painfully slow recovery and thick distorting glasses.

They recognised the status quo as unacceptable. Naming the dangers to build a sense of urgency, they created a burning platform for change; beginning the work of guiding their profession toward the other side of the canyon.

Today's leaders face a similar challenge. When introducing new models of care, the first task is not implementing change but helping colleagues recognise why change is necessary.

### Step 2: build a guiding coalition

Ridley’s coalition began in a parked car in Cavendish Square. After treating RAF pilots during World War II, he noticed acrylic cockpit fragments lodged in their eyes caused little inflammation. He called his friend John Pike, the optical scientist at Rayner, a modest family-run spectacle maker. The two met discreetly after work. Ridley shared an audacious idea: to implant a permanent lens inside the human eye avoiding the need for heavy glasses. Sitting in Ridley’s car, they devised the principles of a new operation.

Most surgeons dismissed the concept; some openly mocked it. But Pike listened. In Rayner’s Brighton workshop, they sketched prototypes and shaped the world’s first intraocular lens by hand: the first plank in the bridge across the canyon.

Kelman’s coalition formed in more unexpected circumstances. After years of trying to achieve small-incision surgery, he found himself at the dentist, where an ultrasonic tool was used to remove calculus. Watching it vibrate, he realised a cataract could be broken up in the same way, removing the need for large incisions. His dentist, Mr. Kuhn, became part of an unlikely coalition that helped him prototype phacoemulsification, an ultrasound-based technique that allows cataracts to be removed through tiny incisions.

A guiding coalition turns a single idea into a movement; it is the leverage that allows one person’s vision to become collective action.

Modern innovation demands similarly diverse coalitions. Successfully implementing AI, for example, requires clinicians, data scientists, patients, informaticians, regulators and industry working towards a shared goal.

### Step 3: form a strategic vision

Visionaries see beyond assumed boundaries. Ridley and Kelman not only recognised the tigers on their side of the canyon; unlike their contemporaries they also imagined what lay across the divide. On the far side they saw food and safety: small incisions, rapid recovery and vision restored to natural clarity. Ridley imagined replacing the lens with a prosthesis, reversing centuries of thinking that surgery was only about removing things. Kelman imagined working through a tiny incision, long before minimally invasive surgery was a concept.

These were daring futures that no one else appreciated. But they could. And they understood a crucial truth of change leadership: a vision only becomes powerful when it is communicated boldly, repeatedly and in many forms – until others believe it too.

How they achieved this would soon prove as revolutionary as the vision itself.

## Engage and enable others

### Step 4: enlist a volunteer army

In 1966, Ridley co-founded the International Intra-Ocular Implant Club (IIIC)^8^. At a time when prominent senior ophthalmologists rejected intraocular lenses, the IIIC became a safe space to share ideas. Ridley had made the why clear: a future without thick distorting glasses. The how emerged from the group itself.

Within the IIIC, surgeons compared techniques, improved lens designs, discussed failures and proposed solutions none could have created alone. Ridley didn’t tell them how to cross the canyon; he simply showed them why the far side mattered.

This is a central lesson in change leadership: when you give people a powerful why, they will often discover the how themselves, and what they create together exceeds anything a single leader could imagine.

### Step 5: enable action by removing barriers

Both men removed structural barriers to change. Kelman knew that even the most compelling vision would go nowhere if surgeons felt unsafe to try it. Phacoemulsification required a microscope that most surgeons had never used. The psychological barrier was high.

To lower that barrier, he ran intensive hands-on courses in New York. He refined the design of the machine, so early adopters did not have to fight unreliable technology. By removing the barriers, he made the crossing feel not only achievable but safe.

Today's barriers are often different but no less significant: poor interoperability, limited digital infrastructure, concerns about algorithmic bias, uncertainty around regulation and a lack of time or training to adopt new technologies. The role of leaders is to create the conditions in which people feel safe, supported and equipped to achieve widespread adoption.

### Step 6: generate short-term wins

Ridley and Kelman understood that visible wins are the most persuasive force in shifting sceptical minds.

Ridley kept his work secret until he had successful outcomes. When he stepped into the spotlight at the Oxford Ophthalmological Congress, he did not rely on slides; he brought a patient with 6/6 unaided vision after lens implantation. Many senior surgeons refused to even look into the patient’s eye, yet the demonstration planted a seed that could not be uprooted: the new way worked.

Kelman took a different route. Confident in phacoemulsification’s dramatic visual recovery, he showcased it through live surgeries. In 1975, he appeared on The Tonight Show, explaining his technique to millions and playing his saxophone on air. It was unorthodox, but it worked. Patients understood it. Surgeons could not ignore it. Kelman’s theatre list filled overnight, and the momentum became impossible to dismiss.

Early wins became living proof, and proof became belief.

## Implement and sustain

### Step 7: sustain acceleration

Acceleration happens when leaders multiply their influence through others.

In Europe, Eric Arnott became a catalyst. After training with Kelman, he returned determined to spread phacoemulsification across the continent, buying early machines and running a rapid series of live-surgery courses. In one whirlwind tour, Arnott and Kelman demonstrated the technique in five cities over ten days. Each course strengthened the bridge and brought more surgeons across the divide.

Similarly, Keiki Mehta trained under Ridley at Moorfields and carried the emerging world of intraocular lenses to India with him. He built training centres, embedding lenses into routine practice and adapted the surgery for low-resource settings; bringing millions closer to the far side of the canyon.

Modern transformation similarly depends on clinical champions and networks that enable learning to spread beyond individual organisations.

### Step 8: institute change

Change becomes permanent only when going back to old ground becomes impossible. Once you’ve crossed the bridge, it must be removed so there is no return. That is precisely what happened in the final phase of the cataract revolution.

By the 1990s, WHO guidelines on intraocular lenses provided global guidance, dispelling lingering scepticism. Professional bodies worldwide endorsed phacoemulsification as the gold standard.

What began as a daring innovation became the only acceptable way to practise. The old methods became socially untenable; no longer a matter of preference, but of safety and ethics. The profession had crossed the canyon, and the bridge behind them had been dismantled.

## Conclusion

The evolution of cataract surgery from couching’s crude restoration of light, is one of the clearest illustrations of crossing the canyon of change.

Change is never easy. Ridley and Kelman faced hostility and ridicule, yet by following the principles captured in Kotter’s model, they overturned centuries of surgical dogma. With the first intraocular lens, Ridley proved that a prosthesis could safely remain inside an organ, a conceptual leap that paved the way for implantable technologies that now define modern medicine.

Ridley was eventually knighted and Kelman received the US National Medal of Technology. And what of Rayner, the tiny optician’s shop where the first intraocular lens was handcrafted? It grew into a global company exporting millions of lenses worldwide each year^9^.

But their true legacy is not honours or international recognition. It is the millions who opened their eyes after surgery and saw the world clearly again. It is the courage to imagine a future others could not yet imagine.

As healthcare faces a new wave of technological breakthroughs, the leadership challenge remains unchanged. Innovation alone does not transform healthcare. People do. The leaders who create urgency, build coalitions, inspire belief and support others to cross the canyon will determine whether today's breakthroughs become tomorrow's standard of care.

Ridley and Kelman's journey shows that real leadership builds bridges that render the old ground obsolete; carrying generations safely forward long after the builders themselves have gone. Yet every bridge, however transformative, leads to another canyon waiting to be crossed.

## Ethics approval and consent to participate

Ethical approval and informed consent were not required, as this article is based solely on published literature and does not involve human participants, human data or human tissue.

## CRediT authorship contribution statement

**Sabah Janjua:** Conceptualization, Project administration, Writing – original draft, Writing – review & editing. **Jonathan Benger:** Conceptualization, Supervision, Writing – review & editing.

## Funding

This work did not receive any specific grant from funding agencies in the public, commercial, or not-for-profit sectors.

## Declaration of Generative AI and AI-assisted technologies in the writing process

During the preparation of this work, the authors used Perplexity AI to support literature searching. After using this tool, the authors critically reviewed, revised and edited all content as needed and take full responsibility for the content of the publication.

## Declaration of competing interest

The authors declare that they have no known competing financial interests or personal relationships that could have appeared to influence the work reported in this paper.
